# Optogenetically enhanced pituitary corticotroph cell activity post-stress onset causes rapid organizing effects on behaviour

**DOI:** 10.1038/ncomms12620

**Published:** 2016-09-20

**Authors:** Rodrigo J. De Marco, Theresa Thiemann, Antonia H. Groneberg, Ulrich Herget, Soojin Ryu

**Affiliations:** 1 Developmental Genetics of the Nervous System, Max Planck Institute for Medical Research, Jahnstr 29, 69120 Heidelberg, Germany; 2 Focus Program Translational Neuroscience, Johannes Gutenberg University Medical Center, Langenbeckstr 1, 55131 Mainz, Germany

## Abstract

The anterior pituitary is the major link between nervous and hormonal systems, which allow the brain to generate adequate and flexible behaviour. Here, we address its role in mediating behavioural adjustments that aid in coping with acutely threatening environments. For this we combine optogenetic manipulation of pituitary corticotroph cells in larval zebrafish with newly developed assays for measuring goal-directed actions in very short timescales. Our results reveal modulatory actions of corticotroph cell activity on locomotion, avoidance behaviours and stimulus responsiveness directly after the onset of stress. Altogether, the findings uncover the significance of endocrine pituitary cells for rapidly optimizing behaviour in local antagonistic environments.

## Abstract (web summary)

Behavioural adaptations in response to stress are thought to be regulated by rapid neurotransmitter action, followed by slower hormonal signalling. Here, using optogenetic approaches, the authors find corticotroph cell populations are capable of rapidly modulating avoidance behaviours immediately after the onset of stress.

## Graphical abstract

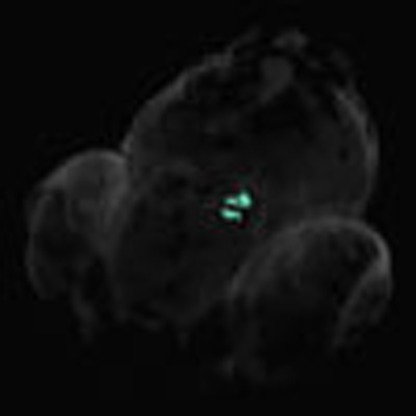

Reversible phenotypic adaptations enable animals to adjust to local environmental changes, and usually consist of rapid physiological or sensory changes exerting meaningful influences on behaviour. Hormones govern these changes, just as the environment influences humoural responses. Antagonistic environments stimulate a range of tightly regulated processes jointly referred to as the stress response^1^. They serve adaptation and survival, and involve changes in physiology and behaviour on multiple timescales. The response relies heavily on the hypothalamic–pituitary–adrenal (HPA) axis, responsible for rapid and long-term processes counteracting adversity^2^. The pituitary gland is thus the bridge between nervous and hormonal control systems, and regulates vital functions under hypothalamic control both normally and in response to stress^3^.

The onset of stress elicits a modulation of anterior pituitary hormone-secreting cells through hypophysiotropic neurohormonal actions, which eventually leads to the expression and release of pituitary hormones into the circulation^4^. Stressors for instance prompt neurons in the paraventricular nucleus of the rostral hypothalamus to release neurotransmitters like corticotropin-releasing hormone (CRH), the primary stimulator of the HPA axis^5^. CRH binds to the CRH receptor (CRHR) type 1 in pituitary corticotrophs^6^ and stimulates cAMP and Ca^2+^ increase preceding hormone secretion^7^. Corticotrophs comprise a fraction of the anterior pituitary and express proopiomelanocortin (POMC), the precursor of adrenocorticotropic hormone (ACTH). CRH is thus the most potent ACTH secretagogue, and stimulates corticotrophs to release ACTH which in turn induces the adrenal cortex to produce and release cortisol^5^. Stress can be defined empirically as any condition eliciting the rapid release of ACTH followed by that of cortisol. Because cortisol release is consistently anchored to ACTH release, pituitary corticotrophs govern the activity of the pituitary–adrenal unit, and consequently, the long-term fate of the stress response. Immediately after the onset of stress, however, their role in regulating reversible phenotypic adaptations is far less understood.

For the stress response to be effective as an adaptive response to the environment, actions of hypothalamic and anterior pituitary neuropeptides must be temporally and spatially coordinated. How these modulators interact with each other to regulate processes within particular time windows is unclear^8^. Furthermore, the contribution of distinct modulators to coping behaviour has been difficult to dissect because the onset of stress affects several neurotransmitters and hormonal systems simultaneously. For instance, in addition to its endocrine functions, CRH may act as a neuromodulator in extrahypothalamic circuits, producing a range of autonomic, electrophysiological and behavioural effects in response to stress, including increased stimulus responsiveness^9^,^10^. ACTH and glucocorticoids like cortisol have central and peripheral targets, and can exert rapid actions as well. Fast glucocorticoid effects on neural activity in mammals have been documented in multiple brain areas^11^,^12^,^13^, including the hippocampus^14^,^15^, basolateral amygdala^16^ and the hypothalamus^17^,^18^,^19^. Similarly, behavioural correlates of ACTH and glucocorticoid injections occurring within minutes to tens of minutes post injection have been reported^20^,^21^,^22^,^23^,^24^. Despite these advances, the notion that pituitary corticotrophs may contribute to reversible phenotypic adaptations has so far been based on hormone injections and varying, usually delayed, measures of dissimilar end points. Due to the limited accessibility of the hypothalamus and pituitary and the coupled release of hypothalamic and pituitary neuropeptides, it has been difficult to selectively modify endogenous activity levels in pituitary corticotroph cells so as to identify their rapid modulatory actions on behaviour.

We address this question in larval zebrafish (*Danio rerio*) using optogenetics and find that optogenetic enhancement of corticotroph cell activity, immediately after the onset of stress, is sufficient to induce reversible phenotypic adaptations in very short timescales. Zebrafish larvae are chosen because their hypothalamic–pituitary–interrenal (HPI) axis is homologous to the HPA axis^25^, their genetic amenability and transparent body make them ideal for non-invasive optogenetics, and their small size allows for the continuous measurement of behaviour with full control of the environment, including the onset of acutely threatening stimuli. The activity of pituitary corticotrophs can be modified by expressing *Beggiatoa* photo-activated adenylyl cyclase (bPAC)^26^,^27^ specifically in these cells^28^. Using light of different wavelengths as both a potent stressor and a means for wavelength-dependent optogenetic control of selective cell activity, cAMP-mediated Ca^2+^ levels in pituitary corticotroph cells can be amplified, preceding a higher hormone release and eventually resulting in over-elevated whole-body cortisol. A set of novel assays furthermore allows the specification of the ensuing modulatory actions on locomotion, stressor avoidance and stimulus responsiveness. Altogether, our findings provide direct evidence that pituitary corticotroph cells can rapidly modulate avoidance behaviours on the onset of stress.

## Results

### Optogenetic manipulation of pituitary corticotroph cells

**Figure 1:**
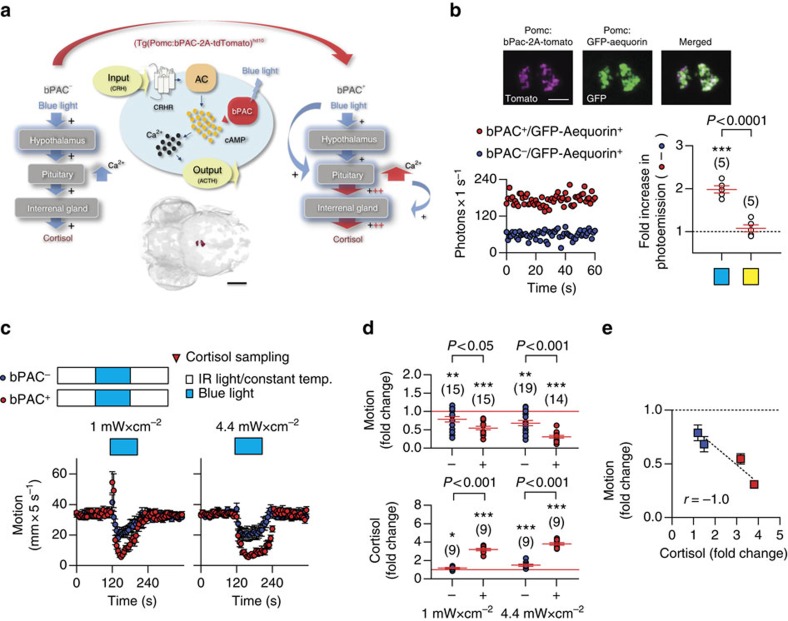
Blue-light-stimulated bPAC^+^ larvae show strengthened stress reactions. (**a**) Schematic showing the expected amplification of cAMP-dependent hypothalamic signalling and Ca^2+^-dependent hormone release in pituitary corticotrophs in presence of bPAC. bPAC^+^ larvae are expected to show an amplified level of pituitary corticotroph cell activity and subsequent cortisol release in response to blue light, as compared with bPAC^*−*^ larvae. Bottom, dorsal view of bPAC expression in pituitary corticotrophs as detected by fused tdTomato fluorescence. Scale bar, 500 μm. Modified from ref. 28. (**b**) The Ca^2+^-sensitive photoprotein GFP-Aequorin is expected to produce higher Ca^2+^-dependent photo-emission in corticotroph cells as a result of a blue-light-dependent bPAC activation. Top, Co-expression (right) of fluorescent tdTomato (left) and GFP (center). Scale bar, 50 μm. Bottom left, Representative post-blue-light Ca^2+^-dependent photo-emission time curves of bPAC^+^/GFP-Aequorin^+^ and bPAC^*−*^/GFP-Aequorin^+^ larvae pre-incubated in the Aequorin substrate coelenterazine, whose oxidation results in photon emission. Bottom right, After a brief exposure to blue light (blue square), bPAC^+^/GFP-Aequorin^+^ larvae on average showed a twofold increase in photo-emission, that is, area under the photons s^*−*1^-time curve, as compared with bPAC^*−*^/GFP-Aequorin^+^. This was not the case with a yellow-light exposure (yellow square), reflecting a blue-light-dependent Ca^2+^ increase as a blue-light-specific response (***P<0.001 after one-sample *t*-tests against a fold change of ‘1' (dashed line), *P* value after a two-tailed *t*-test). (**c**) Top, groups and protocol for testing responses of briefly dark-adapted bPAC^+^ and bPAC^*−*^ larvae to a squared pulse of blue light. Bottom, individual motion (mean±s.e.m., in mm per 5 s) before, during and after a squared pulse of blue light of either low (left) or high (right) power. (**d**) Blue-light-mediated motion (top) and cortisol (bottom) change in bPAC^+^ and bPAC^*−*^ larvae as a function of light power. *P<0.05, **P<0.01, ***P<0.001 after one-sample *t*-tests against a fold change of ‘1' (red lines), *P* values indicate results of Bonferroni's tests after a two-way ANOVA. (**b**,**d**) Sample size in parentheses. (**e**) Correlation between motion and cortisol fold change from **c** and **d**, respectively.

Larval zebrafish are highly sensitive to light and react to a sudden dark-to-light transition with a brief period of increased locomotion immediately after the light onset, followed by reduced locomotion during the light period and recovery of baseline locomotion after the light offset^28^. These locomotor reactions closely mimic those of a pH drop, a known potent stressor in fish (Supplementary Fig. 1a). In briefly dark-adapted larvae, a squared pulse of either blue or yellow light not only elicits locomotor reactions, but also increases whole-body cortisol in a graded manner, depending on light power (per unit of area) and exposure time (Supplementary Fig. 1b, see also ref. 28). As a result, the overall locomotion reduction occurring during the light period correlates well with light power and the ensuing cortisol level (Supplementary Fig. 1c). Considering the rapidly increased levels of cortisol as the end point of the HPI axis activation, these observations indicated that a sudden dark-to-light transition can act as a threatening stimulus that is sufficient to activate all elements of the stress response (see also Supplementary Discussion and Supplementary Movie 1). To identify rapid correlates of enhanced endocrine pituitary cell activity on stress onset, we targeted the expression of bPAC to corticotroph cells using a fragment of the POMC promoter^29^ (Fig. 1a, Supplementary Movie 2).

The activation of bPAC is blue-light-specific due to the protein's BLUF (blue-light receptor using flavine adenine dinucleotide)-type light sensor domain^26^,^27^. Previous work showed that the injection of bPAC mRNA into one-cell stage embryos causes a blue-light-dependent elevation of whole-body cAMP at 1 day post fertilization (d.p.f.)^28^. bPAC in corticotroph cells is thus expected to prompt an increased cAMP production specifically in response to blue light. In pituitary corticotrophs, cAMP increase leads to enhanced intracellular Ca^2+^ regulating hormone secretion^30^(see also Supplementary Discussion). We therefore monitored corticotroph cell activity in larvae expressing bPAC in pituitary corticotrophs (*Tg(Pomc:bPAC-2A-tdTomato)*^*hd10*^ larvae^28^, from now on bPAC^+^ larvae) by targeting the Ca^2+^-sensitive photoprotein GFP-Aequorin^31^ to the same cells, thus generating double-transgenic larvae co-expressing bPAC and GFP-Aequorin (*Tg(Pomc:bPAC-2A-tdTomato*^*hd10*^*, Pomc:GFP-Aequorin*^*hd20*^) larvae, from now on bPAC^+^/GFP-Aequorin^+^ larvae; Fig. 1b, top and Supplementary Movie 3). After a brief exposure to blue light, bPAC^+^/GFP-Aequorin^+^ larvae on average showed a twofold increase in photo-emission as compared with their negative siblings (*Tg(Pomc:bPAC-2A-tdTomato*^*hd10*^)^*−*^ larvae, from now on bPAC^−^ larvae) also co-expressing GFP-Aequorin (bPAC^−^/GFP-Aequorin^+^ larvae). This was not the case with an exposure to yellow light, further highlighting the over-elevated Ca^2+^ level increase as a blue-light-specific phenomenon (Fig. 1b, bottom; two-tailed *t*-test, 1 mW cm^*−*2^, bPAC^+^/GFP-Aequorin^+^ versus bPAC^−^/GFP-Aequorin^+^, t(8)=7.6, *P*<0.0001, one-sample *t*-test against a fold change of ‘1', bPAC^+^/GFP-Aequorin^+^, t(4)=11.7, *P*=0.0003, bPAC^−^/GFP-Aequorin^+^, t(4)=0.9, *P*=0.42).

In pituitary corticotrophs, the cAMP increase downstream of CRHR activation precedes the release of ACTH^30^. The stimulation of bPAC by blue light, responsible for the enhanced levels of Ca^2+^, is thus expected to amplify the level of circulating ACTH and, as a consequence, that of whole-body cortisol (Fig. 1a) since the ACTH receptor (melanocortin receptor type 2 (MC2R)) is expressed in the interrenal gland (Supplementary Fig. 2, see also ref. 32). As expected, blue light rapidly led to a transient, wavelength-specific, light power-dependent state of over-elevated cortisol in bPAC^+^ larvae^28^ (see also Fig. 1d). Altogether, these results indicated that, for bPAC^+^ larvae, light can be used as a highly controllable stressor as well as a means to enhance the level of pituitary corticotroph cell output immediately after the onset of stressor exposure.

### Strengthened endocrine and locomotor reactions

Following several minutes of dark-adaptation and having been subsequently exposed to a pulse of blue light, bPAC^+^ and bPAC^*−*^ larvae showed similar levels of baseline locomotion and whole-body cortisol before the light pulse (Supplementary Fig. 3, see also ref. 28). Both groups showed a light-mediated locomotion reduction, but the reduction was significantly greater in bPAC^+^ larvae (Fig. 1c). Similarly, in response to light of low power, both groups showed a brief period of increased locomotion immediately after the light onset, and again a slight increase before the light offset. The magnitude of these locomotor reactions was also greater in bPAC^+^ larvae (Fig. 1c, left). To compare light-mediated changes in locomotion between bPAC^+^ and bPAC^*−*^ larvae, we calculated ‘motion change' as the ratio between motion during and before the light pulse for each larva, with motion being measured as the integral of distance swum every 10 ms for equal time periods during and before the light pulse. Motion change values across groups confirmed that the higher the light power, the higher the magnitude of the light-mediated locomotion reduction, and that bPAC^+^ larvae displayed greater reductions than bPAC^−^ larvae (Fig. 1d, top, two-way analysis of variance (ANOVA), genotype factor: F(1,59)=24.3, *P*<0.0001, light power factor: F(1,59)=7.5, *P*=0.008, genotype × light power factor: F(1,59)=1.1, *P*=0.30, followed by *post hoc* comparisons). Similarly, so as to compare the light-mediated HPI axis activation level of bPAC^+^ and bPAC^−^ larvae, we calculated ‘cortisol change' as the ratio between whole-body cortisol before and after (2 min) the light pulse for each group. Cortisol change values across groups showed that the magnitude of the light-mediated cortisol change also depended on light power: the higher the power, the higher the change, which was consistently greater in bPAC^+^ larvae, thereby confirming previous results^28^ (Fig. 1d, bottom, two-way ANOVA, genotype factor: F(1,32)=317.6, *P*<0.0001, light power factor: F(1,32)=16.1, *P*=0.0003, genotype × light power factor: F(1,32)=1.1, *P*=0.30, followed by *post hoc* comparisons). Thus, the blue-light-mediated locomotor reduction and cortisol increase in bPAC^+^ and bPAC^−^ larvae were negatively correlated to each other (Fig. 1e, Spearman's rank correlation, *P*<0.0001, *r*=−1.0). To rule out the possibility that the observed differences in light-mediated motion and cortisol change between bPAC^+^ and bPAC^−^ larvae were due to non-specific effects of light on other agents of the stress response, we performed additional experiments following the same protocols but now substituting blue for yellow light, as bPAC activation is restricted to blue-light illumination^26^,^27^. Yellow light yielded effects on motion and cortisol change similar to those of blue light, but did not allow for a further group distinction between bPAC^+^ and bPAC^−^ larvae as it was the case with blue light (Supplementary Fig. 4). In sum, only blue light strengthened locomotor reactions and further increased whole-body cortisol in bPAC^+^ larvae. Also, for bPAC^+^ larvae, the magnitude of the blue-light-dependent elevation of cortisol increased with light power and exposure time. We concluded that the enhanced activity level of pituitary corticotroph cells was sufficient for inducing differences in endocrine and locomotor reactions to light between both groups of larvae.

### Corticotroph cells aid in rapidly coping with threats

**Figure 2:**
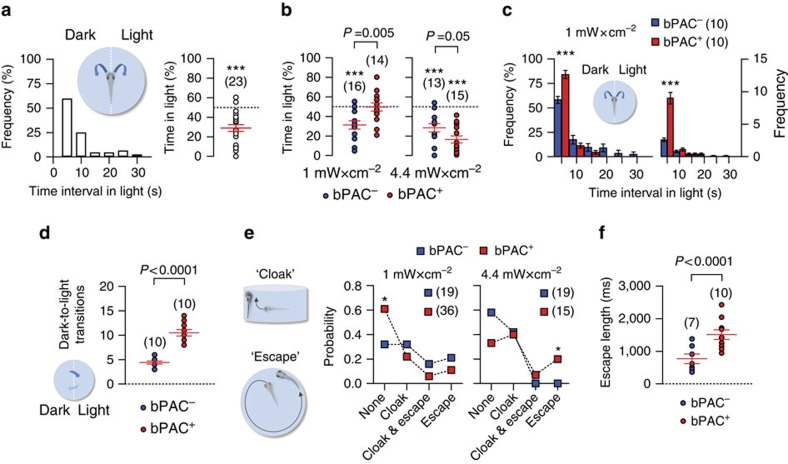
Enhanced pituitary corticotroph cell activity causes rapid adjustments of stressor avoidance. (**a**) Inset, configuration for assessing light avoidance in freely swimming larvae. Left, distribution of time intervals under unfiltered light during a 120 s period of blue-light illumination. Right, cumulative time spent under blue light by individual wild-type larvae over a 120 s illumination period (in %) relative to a maximum of 120 s. (**b**) Cumulative time spent under blue light (as in **a**) by individual bPAC^+^ and bPAC^*−*^ larvae for two different light powers; *P* values show results after two-tailed *t*-tests. (**a**,**b**) ***P<0.001 after one-sample *t*-tests against 50%. (**c**) Inset, configuration for assessing light avoidance in freely swimming larvae (as in **a**). Light power, 1 mW cm^*−*2^. Relative (in %) (left) and absolute (right) frequencies of time intervals under unfiltered light (mean±s.e.m.) by individual bPAC^+^ and bPAC^*−*^ larvae during a 120 s period of blue-light illumination. ***P<0.001 after Two-tailed *t*-tests. (**d**), Total number of dark-to-light transitions by individual bPAC^+^ (red) and bPAC^*−*^ (blue) larvae during the 120 s period of blue-light illumination (*P* value after a Two-tailed *t*-test). (**e**), Left, Scheme showing avoidance behaviours elicited by continuous light exposure: ‘cloak' (top), negative geotaxis combined with thigmotaxis and ‘escape' (bottom), a swimming burst. Right, probabilities of responses to blue light in bPAC^+^ and bPAC^*−*^ larvae as a function of light power. *P<0.05 after G-tests. (**f**) Length of escape reactions by individual bPAC^+^ and bPAC^*−*^ larvae during blue-light stimulation. Light power, 1 and 4.4 mW cm^*−*2^. *P* value after a two-tailed *t*-test. (**a**–**f**) Sample size in parentheses.

The light-mediated locomotor reduction occurring in wild-type and bPAC larvae in response to either blue or yellow light pointed to a regulation of locomotion that aids in coping with threatening stimuli by reducing threat exposure. To test this hypothesis, we examined a larva's choice between residing beneath filtered (darkness) and unfiltered light (Fig. 2a, insert, light power, 0 and 1 mW cm^−2^ for darkness and unfiltered blue light, respectively). Briefly dark-adapted (wild-type) larvae actively avoided the incidence of light when offered a choice between darkness and unfiltered light (Fig. 2a, left, frequency distribution of time intervals in light over 120 s, right, cumulative time spent under blue light by individual larvae over a 120 s illumination period (in %) relative to a maximum of 120 s; one-sample *t*-test against 50%, t(22)=6.2, *P*<0.0001). In bPAC siblings, this form of choice depended on bPAC expression, and the wavelength and power of the stimulating light. In response to blue light, bPAC^*−*^ larvae avoided the light irrespective of its power, as indicated by their cumulative time spent in light, and, for a high-light power, bPAC^+^ larvae showed a greater preference for darkness. Contrary to expectations, at a low-light power bPAC^+^ larvae did not spend more time in darkness than in light (Fig. 2b, Two-tailed *t*-test, 1 mW cm^*−*2^, bPAC^+^ versus bPAC^*−*^, t(28)=3.1, *P*=0.005, 4.4 mW cm^*−*2^, bPAC^+^ versus bPAC^*−*^, t(26)=2.2, *P*=0.041, one-sample *t*-test against 50%, 1 mW cm^*−*2^, bPAC^*−*^, t(15)=4.4, *P*=0.0005, bPAC^+^, t(13)=0.08, *P*=0.94, 4.4 mW cm^*−*2^, bPAC^*−*^, t(12)=5.3, *P*=0.0002, bPAC^+^, t(14)=9.1, *P*<0.0001). However, a closer look into random samples of equal size of the low-power data indicated that bPAC^+^ larvae not only spent overall shorter intervals in light during the 120 s period (Fig. 2c, Two-tailed *t*-tests, right, t(18)=4.8, *P*<0.0001, left, t(18)=7.0, *P*<0.0001), indicative of a strengthened light avoidance, but also moved more frequently in the darkened half of the chamber, as indicated by the fact that they transitioned to the illuminated half much more frequently than bPAC^*−*^ larvae (Fig. 2d, two-tailed *t*-test, t(18)=8.6, *P*<0.0001). Clearly, this enhanced locomotor activity in the darkened half of the chamber persisted during the 120 s period in spite of the several light exposures on the illuminated side. Since the number of dark-to-light-transitions is representative of their overall activity, these observations altogether pointed to higher stimulus responsiveness and improved coping in bPAC^+^ larvae as compared with their negative siblings.

Without a choice between darkness and light, that is, during constant exposure to light after a brief period of dark-adaptation, both wild-type and bPAC larvae shortened their steps and increased their turns after the onset of either blue or yellow light, thus reducing their overall swum distance. Remarkably, they could also execute two distinctive behaviours. The first one was a combination of negative geotaxis and thigmotaxis (henceforth ‘cloak'; Fig. 2e, top left). The second was a brief swimming burst of high speed (henceforth ‘escape'; Fig. 2e, bottom left). These two behaviours appeared hierarchically organized; in so far as that the energetically less-costly cloak was more frequent than escape (Supplementary Fig. 5) and both actions could occasionally be observed alternating within a short time period. In bPAC siblings, the probability of these behaviours also depended on bPAC expression, and the wavelength and power of the stimulating light. bPAC^+^ larvae responded to blue light of low power primarily with modified locomotion (shortened steps and increased turns), in line with the more frequent dark-to-light transitions when offered a choice between filtered and unfiltered low-power light (Fig. 2e, centre, G-test, *P*=0.003). As a result, they on average showed less cloaks and escapes than bPAC^−^ larvae. By contrast, in response to blue light of higher power, bPAC^+^ larvae displayed more frequent and lengthy escapes than bPAC^*−*^ larvae (Fig. 2e, right, G-test, *P*=0.0004, Fig. 2f, two-tailed *t*-test, bPAC^+^ versus bPAC^*−*^, t(15)=3.5, *P*=0.004; Supplementary Movies 4 and 5), in line with a greater preference for darkness (less time spent in light) when offered a choice between filtered and unfiltered light of high power. Yellow light did not yield a similar distinction between bPAC siblings (Supplementary Fig. 6). Both bPAC^+^ and bPAC^*−*^ responded in the same manner (as the bPAC^*−*^ with blue light), with a preference for darkness when offered a choice, irrespective of light power and bPAC expression (Supplementary Fig. 6a). Similarly, for light of low and higher power, both groups showed no difference concerning the frequency of cloaks and escapes, as well as escape length (Supplementary Fig. 6b,c).

On the underlying assumption that a larva's goal of reducing the incidence of light is mediated by stimulus salience and strength on the one side, and their capacity for threat detection and mobilizing resources on the other, bPAC^+^ larvae appeared to be better equipped to handle a threatening environment by first seeking avoidance of the threat cue altogether, and if not given a choice, by directly investing more resources into elaborate cloaks and escapes. Thus, bPAC^+^ larvae showed an affinity towards modified locomotor patterns at a low-light power, and cloak and escape responses at a high-light power, coupled with greater avoidance if given a choice. Cloak and escape presumably require more resources to actively sustain, making these responses more demanding. For low-light powers, bPAC activation might better prepare larvae to tolerate low-level threats, whereas at a high-light power they will still be able to allocate a vaster capacity towards the potentially more efficient escape.

### Increased stimulus responsiveness under stress

**Figure 3:**
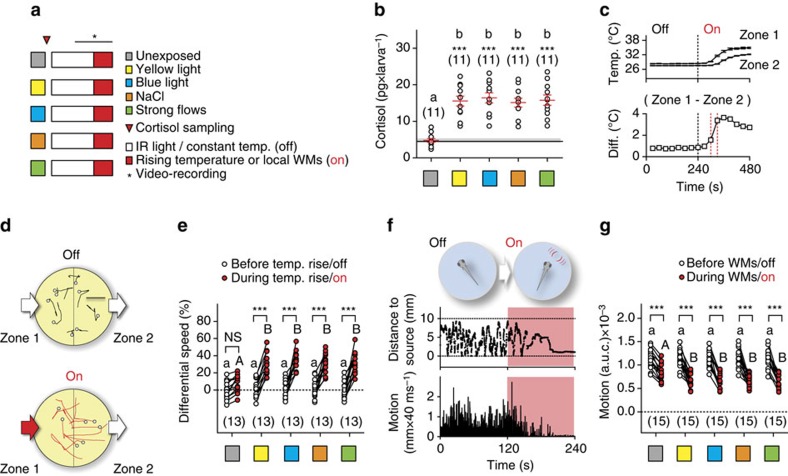
Increased stimulus responsiveness follows stressor exposure in wild-type larvae. (**a**) Groups for testing the effect of stressor exposure on larval responsiveness to rising temperature and WMs. (**b**) Whole-body cortisol in wild-type larvae for the groups in **a**. Letters indicate results from *post hoc* tests after a one-way ANOVA (*P*<0.01) and asterisks (*P*<0.001) from one-sample *t*-tests against basal cortisol in controls (black line). (**c**) Increasing the temperature of the flowing medium raises the temperature difference (ΔT) between the opposing inlet (higher temperature, zone 1) and outlet (lower temperature, zone 2) of the swimming chamber. Top, mean temperature (±s.e.m.) in zones 1 and 2 as a function of time. Bottom, ΔT as a function of time. (**d**) From top to bottom, representative 1 s swim paths from pre-exposed larvae showing increased speed and turns near zone 1 as ΔT increases. White dots indicate start positions. Scale bar, 2.5 mm. (**e**) Differential speed (the difference between swim velocity (mm per 40 ms) in zone 1 and 2 (in %)) for the groups in **a** before and after the onset of temperature rise. (**f**) Schematic showing the stimulation procedure (top) and representative traces of a larva's distance to stimulus source (middle) and swim velocity (bottom) before and after the onset of WMs. Red backgrounds depict the period of WMs. (**g**) Motion level, that is, area under the swim velocity-time curve over 120 s, in wild-type larvae before and during WMs. (**e**,**g**) Letters and asterisks indicate results from *post hoc* comparisons after a two-way repeated measures ANOVA (*P*<0.001). (**b**,**e**,**g**) Sample size in parentheses.

To test if stressor exposure leads to increased stimulus responsiveness in larval zebrafish, we developed two novel behavioural assays with a focus on measuring responsiveness to different sensory stimuli, and assessments of both avoidance and attraction. In addition, to better pinpoint effects invariant to stressor identity, we used three different stimulations eliciting avoidance reactions and cortisol increase in a stimulus intensity-dependent manner: exposure to either light, hyperosmotic medium or strong hydrodynamic flows (Fig. 3a, see also refs 28, 33). To compare the level of HPI axis activation produced by these stimulations, we first measured whole-body cortisol in wild-type larvae directly after stressor exposure. Cortisol values across groups of pre-exposed (stressed) larvae had nearly equal distributions and were, on average, ∼230% higher than those of unexposed larvae (Fig. 3b, one-way ANOVA, F(4,54)=16.4, *P*<0.0001, followed by *post hoc* comparisons, One-sample *t*-test against basal cortisol, yellow light: t(10)=8.0, *P*<0.0001, blue light: t(10)=8.5, *P*<0.0001, NaCl: t(10)=8.6, *P*<0.0001, strong flows: t(10)=7.9, *P*<0.0001).

Next, in the first assay, we determined the link between stressor exposure and the subsequent reaction of a freely swimming larva encountering a higher surrounding water temperature. We monitored the movements of single larvae swimming in darkness in a small, cylindrical chamber with an opposing in and outlet before and after a sharp temperature increase of the flowing medium, which, owing to the layout of the chamber and heat conduction, led to a significant temperature difference between the inlet (high temperature, zone 1) and outlet (low temperature, zone 2) area (Fig. 3c, see also Supplementary Fig. 7c and see the ‘Methods' section). After the onset of temperature rise, larvae entering the high-temperature area reacted to an increasing surrounding temperature with fast turns and greater swim velocity (Fig. 3d). To quantify their overall response, we measured ‘differential speed' (ΔS) as the difference (in %) between the swim velocity (mm × (40 ms)^*−*1^) in zone 1 and 2 for each larva, or ((swim velocity in zone 1−swim velocity in zone 2)/swim velocity in zone 2) × 100, before (30 s) and after (60–90 s) the onset of the temperature rise (see also the ‘Methods' section). The distribution of ΔS values across groups revealed that unexposed larvae displayed only slightly modified locomotion patterns in zones 1 and 2 after the onset of temperature rise, whereas larvae pre-exposed to stressors had nearly equally distributed ΔS values that were, on average, 316% higher than those of unexposed larvae (Fig. 3e, top, two-way repeated measures ANOVA, time factor: F(1,60)=246.2, *P*<0.0001, treatment factor: F(4,60)=5.6, *P*=0.0006, time × treatment factor: F(4,60)=7.0, *P*=0.0001, followed by *post hoc* comparisons).

In the second assay, we applied subtle hydrodynamic stimuli to similar groups of wild-type larvae (Fig. 3a). Hydrodynamic sensing provides fish with various benefits, ranging from object detection to sensing conspecifics^34^. Larval zebrafish, for instance, respond to local, non-stressful water motions (WMs) with positive taxis and reduced locomotion^35^. The onset of WMs first leads them to approach the stimulus source and reduce their locomotion gradually; they then remain virtually immobile in the proximity of the source as long as WMs persist (Fig. 3f). This response is highly sensitive to stimulus frequency and strength, sensory background and rearing conditions^35^, and offers an excellent handle for assessing subtle changes in stimulus responsiveness. To quantify a larva's response to WMs, we focused on the WM-induced locomotion reduction and measured integrals of distance swum every 40 ms for equal periods before and during WMs for each larva (see the ‘Methods' section). The distribution of motion values across groups showed responses for pre-exposed larvae that were, on average, 20% greater than those of unexposed larvae, consistent with the idea that increased stimulus responsiveness follows stressor exposure (Fig. 3g, two-way repeated measures ANOVA, time factor: F(1,70)=402.9, *P*<0.0001, treatment factor: F(4,70)=1.0, *P*=0.41, time × treatment factor: F(4,70)=2.2, *P*=0.08, followed by *post hoc* comparisons). Taken together, these results indicated that the various stressors produced positively correlated changes in cortisol, ΔS and motion values.

**Figure 4:**
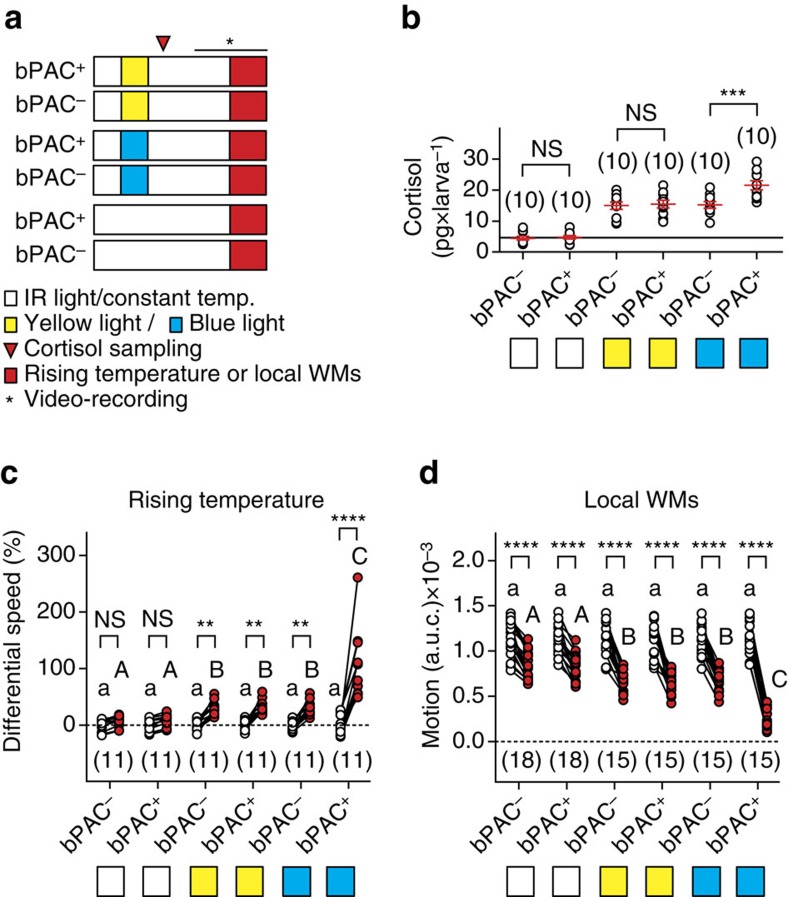
bPAC activation is sufficient to further elevate the level of stressor-mediated stimulus responsiveness. (**a**) Groups for testing the response of bPAC^+^ and bPAC^*−*^ larvae to rising temperature and WMs. (**b**–**d**) Whole-body cortisol, differential speed and motion level (as in Fig. 3b, (**e**,**g**) respectively) for the groups in **a**. Asterisks (***P<0.001, ****P<0.0001) and lower- and upper-case letters (*P*<0.001) indicate results from Bonferroni's tests after a two-way ANOVA (**b**) and two-way repeated measures ANOVAs (**c**,**d**). Sample size in parentheses.

Using bPAC siblings, we then tested if bPAC activation in corticotroph cells influences stressor-mediated levels of stimulus responsiveness. To test this possibility, we determined whether bPAC^+^ and bPAC^*−*^ larvae pre-exposed to either blue or yellow light were equally reactive to increasing water temperature and WMs (Fig. 4a). As expected, both blue and yellow light increased whole-body cortisol in bPAC^+^ and bPAC^*−*^ larvae, and the resulting levels were significantly higher (42%) in bPAC^+^ larvae only after blue-light exposure (Fig. 4b, two-way ANOVA, genotype factor: F(1,54)=7.5, *P*=0.008, treatment factor: F(2,54)=91.9, *P*<0.0001, genotype × treatment factor: F(2,54)=5.3, *P*=0.008, followed by *post hoc* comparisons). Notably, ΔS and motion values showed (a) that unexposed bPAC^+^ and bPAC^*−*^ larvae responded similarly to increasing surrounding water temperature and WMs, (b) that blue and yellow light increased their responses as in wild-type larvae and (c) that bPAC^+^ larvae had much greater responses than bPAC^*−*^ larvae only after blue-light exposure (Fig. 4c,d, two-way repeated measures ANOVA, ΔS, time factor: F(1,60)=117.9, *P*<0.0001, group factor: F(5,60)=11.6, *P*<0.0001, time × group factor: F(5,60)=19.9, *P*<0.0001, motion values, time factor: F(1,90)=747.1, *P*<0.0001, group factor: F(5,90)=9.6, *P*<0.0001, time × group factor: F(5,90)=25.2, *P*<0.0001, followed by *post hoc* comparisons). These results indicated that bPAC activation post-stress onset was sufficient to further elevate the level of stimulus responsiveness.

## Discussion

The difficult *in vivo* accessibility of the hypothalamus and pituitary gland, and the rapid and synchronous release of stress-sensitive modulators have made it difficult to determine the contribution of distinct HPA-axis effectors on immediate reactions to stress. Zebrafish offer a highly *in vivo* accessible and easy-to-handle model for HPA signalling at the same time as a high physiological and neuroanatomical homology to mammals. The zebrafish HPI axis shares conspicuous homologies with the HPA axis in humans^25^ and the larval neurosecretory preoptic-hypothalamic area is also homologous to the hypothalamic paraventricular nucleus in mammals^36^. Notably, the HPI axis matures very early in development from 4 d.p.f. onwards^37^,^38^,^39^ and its principal cell clusters can be visualized and manipulated using genetic tools (Supplementary Movie 1). Furthermore, as in mammals, stressors induce predictable alterations in the locomotor activity and feeding drive of freely behaving larvae^28^,^33^ (see also Supplementary Discussion). Our findings uncover fast organizing effects of pituitary corticotroph cells on behaviour, providing direct evidence that rapid behavioural adjustments on stressor exposure can occur depending on the activity level of pituitary corticotrophs. The results are consistent with literature pointing to joint actions of brain neuropeptides and peripheral hormones^8^ and suggest that a higher level of endocrine pituitary cell activity post onset of stress can yield an advantage in coping with acutely threatening environments.

Although CRH has been shown to elicit rapid behavioural effects^10^, its involvement alone does not account for the differences between bPAC^+^ and bPAC^*−*^ larvae in the range of responses examined here. Experiments using the same protocols but with yellow light failed to produce the same group differences as blue light yielded for the wavelength-specific activation of bPAC in bPAC^+^ larvae. This failure further dissociates general effects of light as a stressor from those specific to bPAC activation, namely enhanced cAMP-mediated Ca^2+^ levels in corticotroph cells preceding a higher level of corticotroph output and eventually resulting in higher whole-body cortisol. Without the bPAC-mediated corticotroph output modification, endocrine and behavioural end points showed similar values between groups for yellow light. Light as a stressor thus fails to account for a distinction between bPAC^+^ and bPAC^*−*^ larvae.

Significant differences between both groups emerged with blue light, in line with the idea that the group differences did not result from the initial activity level of the hypothalamic–pituitary unit, but from downstream processes. We had shown in bPAC^+^ larvae that blue light can be used as both a potent stressor and a means to enhance pituitary corticotroph cell activity, thus prompting a higher cortisol level^28^. The fact that light in this dual role can be accurately controlled is important in understanding how distinct stress modulators influence behaviour immediately after the onset of stress, since the combined effects of HPA-axis effectors can occur at short timescales. Compared with controls, bPAC^+^ larvae showed locomotion changes occurring just seconds after the light onset, and a few seconds later they also showed adjustments in avoidance behaviours, followed by greater stimulus responsiveness after the light offset; clearly, the speed of these effects speaks against innocuous effects of the amplified corticotroph cell activity.

Studies in mammals showed that injections of ACTH and related peptides can alter forms of avoidance^20^,^40^,^41^, stretching and yawning^42^, grooming^43^ and sexually motivated actions^44^; thus a role for pituitary peptides in regulating arousal has been suggested^45^. Evidence also shows that glucocorticoid administration can influence locomotion^22^,^46^, agonistic reactions^47^,^48^,^49^ and courtship^23^,^50^,^51^. These studies relied on injections of exogenous hormones and behavioural measures of varying delays; rapid effects of endogenous effectors from the pituitary–adrenal unit have not been documented before. The change in Ca^2+^-dependent photo-emission and whole-body cortisol gave direct evidence that light activated the HPI axis of the zebrafish larva, prompting the rapid release of corticotroph and steroidogenic interrenal cell products. Our findings thus agree with previous studies and extend those for the first time towards rapid effects of endogenous corticotroph cell products on behaviour.

The blue-light-dependent behavioural adjustments in bPAC^+^ larvae may set in once the activity of either corticotroph or interrenal cells, or both, is enhanced. Active molecules have been ascribed to both cell types. Corticotrophs produce several active peptides resulting from the cleavage and processing of the precursor gene *POMC*, like ACTH, pro-γ-melanocyte-stimulating-hormone (MSH) and β-lipotrophin (LPH). β-LPH is further cleaved to β-endorphin and a proportion of ACTH can be cleaved to α-MSH and corticotrophin-like intermediate peptide^52^. These POMC-derived peptides can lead to behavioural change when injected into rats, and ACTH fragments like ACTH 1–10 and ACTH 4–10, also present in α-MSH, β-MSH and β-LPH, have similar effects on avoidance as ACTH itself^21^. Moreover, ACTH fragments devoid of adrenal function can prompt behavioural changes as well, suggestive of a central role for pituitary-derived peptides that may be transported back to the brain^53^, although central mechanisms underlying rapid effects of pituitary peptides on behaviour have not yet been reported.

In fish, behavioural effects of POMC-derived peptides have been studied primarily in the context of food intake and pigmentation^54^, but not in that of stress. ACTH stimulates the release of steroids from the adrenal cortex via the MC2R. Evidence indicates that the expression of MC2R is restricted to the adrenal cortex, although conflicting results have been reported concerning the role of ACTH in the release of epinephrine and norepinephrine by cells in the adrenal medulla^55^,^56^. The interrenal gland of teleosts does not have a strict separation between cortex and medulla. In larval zebrafish, steroidogenic and catecholaminergic cells are intermingled, and MC2R expression is restricted to steroidogenic cells (Supplementary Fig. 2). In mammals, the adrenal cortex produces glucocorticoids, mineralocorticoids and androgens. In teleosts, steroidogenic cells produce mainly cortisol, responsible for glucocorticoid and mineralocorticoid actions. Ultradian and circadian^57^,^58^ as well as stress-induced glucocorticoid variations have wide-reaching cellular correlates. Glucocorticoid actions in a number of brain regions involve membrane-bound and nuclear forms of mineralocorticoid and glucocorticoid receptors, as well as a yet unidentified G-protein-coupled receptor (reviewed in refs 59,60^59^,^60^). Despite these advances, how glucocorticoids, and hypothalamic and anterior pituitary neuropeptides interact with each other to activate and inactivate stress-sensitive processes across multiple time windows still remains unclear.

In this study, we uncovered reversible phenotypic adaptations attributable to enhanced corticotroph cell activity immediately after the onset of stress. In addition, we added a series of robust assays to a growing repertoire of laboratory tests in larval zebrafish. Genetic tools to modify hypothalamic, corticotroph and steroidogenic cell function are being developed^28^,^61^,^62^ and can be combined with optogenetics, pharmacology and behavioural screens to specify distinct actions of HPA-axis hormones and downstream effectors.

## Methods

### Zebrafish husbandry and handling

Zebrafish breeding and maintenance were performed under standard conditions^63^. Embryos were collected in the morning and raised on a 12:12 light/dark cycle in E2 medium (wild-type and bPAC larvae), or in E3 medium with 0.2 mM N-phenylthiourea (PTU, Sigma-Aldrich, #P7629; GFP-Aequorin larvae), at 28 °C. To avoid unspecific activation of bPAC before the tests, transgenic and control embryos were raised inside custom-made reflective containers covered with 550 nm long-pass filters (Thorlabs, Dachau, Germany; Supplementary Fig. 3a). All experiments were carried out with wild-type or transgenic larvae at 4–6 d.p.f. Tests were performed between 09:00 hours and 18:00 hours, with different experimental groups intermixed throughout the day. Zebrafish experimental procedures were performed according to the guidelines of the German animal welfare law and approved by the local government (Regierungspräsidium Karlsruhe; G-29/12).

### Transgenic lines

*Tg(Pomc:bPAC-2A-tdTomato)*^*hd10*^ fish (bPAC)^28^ were crossed with wild-type fish (cross of AB and TL strains, AB/TL) and their progenies maintained in E2 medium and screened (without anaesthetics) for pituitary-specific tdTomato expression (bPAC^+^), or its absence (bPAC^*−*^), at 3–5 d.p.f., at least 1 day before behavioural or cortisol tests. All screenings were performed using a Leica MZ6 fluorescence microscope. To generate the pomc:GFP-Aequorin construct, an ∼1 kb DNA region, upstream of the start codon of the zebrafish *pomc* gene^29^ was PCR-amplified using the forward primer 5′-GGATTAATCTGCTTTAAGACCTCAATTTTTGAGAC-3′ and the reverse primer 5′-AAAGCTAGCCACTCCCCTCACCATCTCTGAGA-3′. The forward primer included an Ase1 restriction site while the reverse primer included a Nhe1-restriction site. The amplified *pomc* promoter fragment was used to replace the CMV promoter of the pG5A vector^31^, which contains Aequorin fused in frame at the 3′-end of the EGFP with a 5 amino acid linker between GFP and apoaequorin. The recombinant plasmid was injected into one-cell stage AB/TL wild-type embryos at 10 ng μl^−1^ concentration in the presence of 10 ng μl^−1^ Tol2 transposase mRNA and 0.05% phenol red (Sigma-Aldrich, #P3532). The progenies of injected fish were maintained in egg water supplemented with 0.2 mM PTU to prevent pigmentation and screened for pituitary-specific GFP expression at 2–4 d.p.f. A single G0 founder was used to establish a stable F1 family for *Tg(Pomc:GFP-Aequorin)*^*hd20*^. *Tg(Pomc:bPAC-2A-tdTomato)*^*hd10*^ and *Tg(Pomc:GFP-Aequorin)*^*hd20*^ fish were crossed and raised in E3 medium plus 0.2 mM PTU and screened for pituitary-specific co-expression of tdTomato and GFP (bPAC^+^/GFP-Aequorin^+^) or single expression of GFP (bPAC^*−*^/GFP-Aequorin^+^) at 3 d.p.f.

### Staining and microscopy

Riboprobes for the *steroidogenic acute regulatory protein* (*star*)^64^ and *mc2r* were synthesized from linearized plasmids following instructions provided with the digoxygenin-labelling mix (Roche, #11277073910). Whole-mount fluorescent *in situ* hybridization and immunohistochemistry were performed as described elsewhere^65^,^66^, using a primary rabbit antibody labelling tyrosine hydroxylase^67^(1:250) and the secondary anti-rabbit antibody Alexa 647 (Invitrogen, #A-21245, 1:1,000). For imaging, specimens were cleared in 80% glycerol (Gerbu, #2006) in phosphate-buffered saline for 1 h. Confocal stacks were recorded using a Leica SP5 confocal microscope with a Nikon 20 × glycerol objective. *In vivo* images of cell populations forming the three elements of the HPI axis were recorded using specific transgenic expression in stable lines and *in situ* cell type mapping. Transgenic larvae expressing *OtpECR6:RFPcaax*^61^, *pomc:GFP*^29^ and *StAR:GFP*^62^ were embedded in 1% low-melt agar (Roth, #6351) and imaged *in vivo* using a Leica SP5 confocal microscope with a 20 × water objective (see also Supplementary Methods). Stacks were evaluated using Amira 5.4 (FEI Visualization Sciences Group) to create maximum intensity projections and rotation videos, and spatially restricted to the volume of interest, excluding signals from planes in front or behind. Brightness and contrast were adjusted for each channel.

### Setup

Video recordings using blue and yellow light, hydrochloric acid (HCl, Merck, #109063), rising temperature and non-stressful WMs were made under infrared illumination delivered through a custom-made array of infrared-LEDs mounted inside a light-proof enclosure. Custom-made drivers, amplifiers, pulse generators and a TTL control box (USB-IO box, Noldus Information Technology, Wageningen, The Netherlands) allowed computer control of blue- and yellow-light illumination, pH level and mechanosensory stimuli (Supplementary Fig. 7a). Larvae were imaged through infrared-sensitive cameras, at either 25 (ICD-49E B/W, Ikegami Tsushinki Co, Ltd, Japan) or 100 frames s^*−*1^ (Firewire Camera, Noldus Information Technology), with a lens (TV Lens, Computer VARI FOCAL H3Z4512 CS-IR, CBC; Commak, NY, USA) positioned above a cylindrical custom-made swimming chamber (Supplementary Fig. 7b). The complete setup was placed on a vibration-free platform (Newport Corp, Irvine, CA, USA). EthoVision XT 7 software (Noldus Information Technology) was used to monitor the movements of individually swimming larvae. Motion values from video recordings made at 25 and 100 frames s^*−*1^ were expressed as distance swum every 40 (mm per 40 ms) and 10 ms (mm per 10 ms), respectively. The swimming chamber (internal diameter: 10 mm, height: 10 mm) had a transparent bottom and two opposite overtures, inlet and outlet (width: 2.5 mm, height: 400 μm; Supplementary Fig. 7c, top), allowing medium (E2 or E3) to constantly flow at 200 μl min^*−*1^ by means of a peristaltic pump (IPC Ismatec, IDEX Health and Science GmbH, Wertheim, Germany). The chamber also had two cylindrical side channels (internal diameter: 400 μm) opposite to each other opening 200 μm above the transparent glass bottom, with their longest axis oriented at an angle of 30° relative to horizontal (Supplementary Fig. 7c, bottom). One such channel held a thermocouple (TS200, npi electronics GmbH, Tamm, Germany) monitoring the temperature inside the chamber and providing feedback to a control system (PTC 20, npi electronics GmbH; Exos-2 V2 liquid cooling system, Koolance, Auburn, WA, USA) that either kept the flowing medium at 28 °C (±0.1 °C) or increased its temperature rapidly in a highly controlled manner (see below). The second side channel allowed passage of the end of a rigid silica capillary tube, or stimulus source (outer diameter: 350 μm, full length: 25 mm, Polymicro Technologies), submerged ∼400 μm into the chamber's inner medium (depth: 5 mm). The opposite end of the capillary tube was fixed to a multilayer bender actuator (PICMA PL140.10, Physik Instrumente (PI) GmbH+Co. KG, Karlsruhe, Germany) with an operating voltage of 0–60 V, a maximum displacement of ±1,000 μm and an unloaded resonant frequency of 160 Hz. The bender, coupled to a pulse generator, a dual piezo amplifier and a TTL control system, produced unidirectional lateral displacements (of 50 μm and controllable speed) of the capillary's submerged end, creating minute, non-stressful WMs within the chamber. The input voltage applied to the actuator (0.5 V) determined the speed of the capillary's lateral displacements^35^. To monitor a larva's response to a pH drop, a computer-controlled perfusion system (Octaflow, ALA Scientific Instruments, Inc, Farmingdale, NY, USA) injected 2 μl of HCl solution (250 mM) into a cylindrical mixing compartment (internal diameter: 1 mm) situated 10 mm from the inlet of the swimming chamber. The mixing compartment was connected to a reservoir of HCl solution coupled to a computer-controlled solenoid valve via Teflon tubing (internal diameter: 230 μm, outer diameter: 600 μm). TTL signals triggered the opening and closing of the valve (time: 1 s, pressure: 1 p.s.i.), allowing the HCl solution to be well mixed with the flowing medium before reaching the inner chamber. For blue- and yellow-light illumination, a computer-controlled custom LED ring surrounding the camera lens was positioned at a fixed distance above the swimming chamber (Supplementary Fig. 7d). The incident angle of the LEDs allowed for homogeneous illumination of the chamber's inner compartment. Each squared pulse of light of varying duration consisted of 100 ms flashes delivered at 5 Hz. Light power was measured through a hand-held light power meter (Newport Corp, Irvine, CA, USA). All experiments involved single larvae, which moved freely within the chamber and were given an initial time period of 10 min to adapt to the chamber's conditions before tests.

### Stressors

Groups of thirty larvae in 30 mm Petri dishes were exposed to three different protocols based on either blue or yellow light, NaCl or strong hydrodynamic flows and used for cortisol measurement or transferred to the custom-made swimming chamber for behavioural testing. Blue or yellow light: dark-adapted larvae were exposed to a 180 s squared pulse of either blue or yellow light as described above. Light power, 2.8 mW cm^*−*2^. NaCl: larvae were incubated for 10 min in steady-state E2+50 mM NaCl (NaCl_50mM_, Merck, #106404) medium at 28 °C under white-light illumination. They were then washed three times with E2 medium. Strong hydrodynamic flows: larvae were presented with strong flows caused by rapid 1 mm lateral displacements of the end of a rigid silica capillary tube fixed to a multilayer piezo bender actuator (input voltage: 6 V), as described elsewhere^33^. The end of the capillary was submerged 2 mm into the medium at the centre of a 35 mm Petri dish half filled (1.8 ml) with E2 medium (orientation relative to water surface: 90°). Larvae were exposed to 6 stimulation units delivered with an inter-stimulation-interval of 250 ms. Each unit consisted of 99 repetitions of 40 ms lateral displacements. Stimulations were carried out at 28 °C under white illumination.

### Whole-body cortisol

Groups of thirty larvae were immobilized in ice water 120 s after exposure to light, NaCl or strong flows. Control samples were collected after equal handling, omitting stressor exposure. Samples were then frozen in an ethanol/dry-ice bath and stored at −20 °C for subsequent extraction. Cortisol extraction and detection were carried out using a home-made cortisol ELISA protocol, as described elsewhere^68^.

### Recordings

Before recordings, single larvae were kept inside the chamber swimming in E2 medium at 28 °C (±0.1 °C) under infrared light for 10 min. They were then exposed to either blue or yellow light, rising temperature or local WMs and their behaviour was video-recorded as described above.

### Light avoidance

One half of the swimming chamber was covered with a single layer of long-pass filter foil. The resulting light power in the filtered chamber-half was reduced to 0.007–0.012% at 488 nm and 0.003–0.007% at 570 nm, relative to the unfiltered half (2.8 mW cm^*−*2^), as measured with a light power meter (see above). Groups of five larvae were video-recorded while swimming freely for 3 min under infrared illumination only, and then presented with a 120 s pulse of either blue or yellow light. Incidences of a larva entering the illuminated side of the chamber were manually scored for timing and duration. Frequency and cumulative time spent in light were calculated over the course of the 120 s light phase for each larva.

### Definition and scoring of ‘cloak' and ‘escape'

Cloak was defined as a tilted position of the body axis with the head facing up towards the LED ring and the body touching the walls of the chamber, that is, negative geotaxis plus thigmotaxis. Escape reactions were defined as fast swimming bouts along the chamber's wall at speeds that defied valid EthoVision XT 7 tracking because the larva was invisible in multiple subsequent videos frames (frame rate: 100 Hz). The end of an escape bout was defined as the first frame in which the larva had slowed down to traceable speed or halted motion entirely. Occurrence, latency and duration of cloak and escape were scored manually for individual larvae during the entire 120 s of either blue or yellow light in each video.

### Rising temperature

Single larvae were video-recorded for 240 s with the temperature of the flowing medium kept at 28 °C (±0.1 °C). The input of the temperature control system was then stepped up by 10 °C, causing the temperature of the flowing medium to reach 34 °C after 120 s. The rising temperature produced a temperature difference between the inlet and outlet, zones 1 (high temperature) and 2 (low temperature), respectively, which reached its maximum 60–90 s after the onset of temperature increase (300–330 s of the whole recording session), a time window used to measure ‘differential speed'.

### Local WMs

Single larvae in the swimming chamber were video-recorded for 120 s under infrared light and constant temperature. Next, they were presented with 1 ms lateral displacements of the silica capillary tube delivered at 1 Hz (input voltage: 0.5 V) for 120 s, as described elsewhere^35^. Motion before and during stimulation was calculated using the integrals of motion over 120 s.

### Bioluminescence

Coelentrazine (CLNZ; Synchem, #s053) was prepared as a 10 mM stock solution in (2-Hydroxypropyl)-β-cyclodextrin (Tocris, #0708) with 11.25% propylene glycol (Sigma-Aldrich, #398039). The stock solution was diluted to a final working concentration of 40 μM CLNZ in E3 medium. On the evening of 3 d.p.f., bPAC^+^/GFP-Aequorin^+^ and bPAC^*−*^/GFP-Aequorin^+^ larvae were dark-incubated in CLNZ solution at 25 °C; the CLNZ solution was replaced with a freshly diluted stock every 24 h. At 6 d.p.f., they were washed four times in E3 medium and transferred in groups of 7 into a swimming chamber similar to that shown in Supplementary Fig. 7b (with neither in nor outlet) filled with E3 medium only. The chamber remained below the LED ring (as shown in Supplementary Fig. 7d) with its transparent glass bottom fixed 1.5 mm above a photomultiplier tube (Hamamatsu Photonics K.K., Shizuoka, Japan) covered with a custom-made TTL-controlled shutter and mounted inside a light-proof enclosure placed on a vibration-free platform. The shutter remained closed at all times until bioluminescence was measured. CLNZ-incubated larvae were first allowed to swim freely for 240 s and then exposed to a 120 s square pulse of either blue or yellow light (100 ms flashes delivered at 5 Hz, light power: 2.8 mW cm^*−*2^). Photo-emission was measured with the photomultiplier tube at 1 Hz for 60 s immediately after the light offset.

### Statistics

All data are shown as single measurement points or mean and standard error of the mean. We used a random experimental design, Spearman's rank correlations, Student's *t*-tests (two-tailed) for two-group comparisons, one-sample *t*-tests, G-tests and ANOVAs for multiple group comparisons (followed by Bonferroni's *post hoc* tests), or their non-parametric equivalents. Normality was tested using Kolmogorov–Smirnov, Shapiro–Wilk and D'Agostino tests. Analyses were made with EthoVision XT 7 (Noldus Information Technology), Matlab 2009b (MathWorks, Inc, Natick, MA; USA), MS-Excel (Microsoft Corp; Redmond, WA, USA), Prism 5 (Graphpad Software Inc, San Diego, CA, USA), Sigma Plot (Systat Software Inc, San Jose, CA, USA), ImageJ (Freeware) and VirtualDub (Freeware).

### Data availability

The data that support the findings of this study are available from the corresponding authors on request.

## Additional information

**How to cite this article:** De Marco, R. J. *et al*. Optogenetically enhanced pituitary corticotroph cell activity post-stress onset causes rapid organizing effects on behaviour. *Nat. Commun.* 7:12620 doi: 10.1038/ncomms12620 (2016).

## Supplementary Material

Supplementary Information Supplementary Figures 1-7, Supplementary Discussion, Supplementary Methods and Supplementary References.

Supplementary Movie 1 The hypothalamic-pituitary-interrenal (HPI) axis in a zebrafish larva: the neurosecretory preoptic region (blue), the pituitary gland (red) and the interrenal gland (green).

Supplementary Movie 2 3D rotation view of bPAC (magenta) expression in the pituitary of a reconstructed 6 dpf larva.

Supplementary Movie 3 3D rotation view of GFP-Aequorin (G5A, green) and bPAC (magenta) expression in the pituitary of a reconstructed 6 dpf larva.

Supplementary Movie 4 A 4 dpf bPAC^-^ larva's locomotion before, during and after a squared pulse of blue light. Light power, 4.4 mW*cm^-2^.

Supplementary Movie 5 A 4 dpf bPAC^+^ larva's locomotion before, during and after a squared pulse of blue light. Light power, 4.4 mW*cm^-2^.
