# Supplementary material for: Optogenetically enhanced pituitary corticotroph cell activity post-stress onset causes rapid organizing effects on behaviour
Source: Nat Commun. 2016 Sep 20;7:12620. doi: 10.1038/ncomms12620 (PMC5034294; doi:10.1038/ncomms12620)
Supplement: Supplementary Information — Supplementary Figures 1-7, Supplementary Discussion, Supplementary Methods and Supplementary References. [file ncomms12620-s1.pdf]

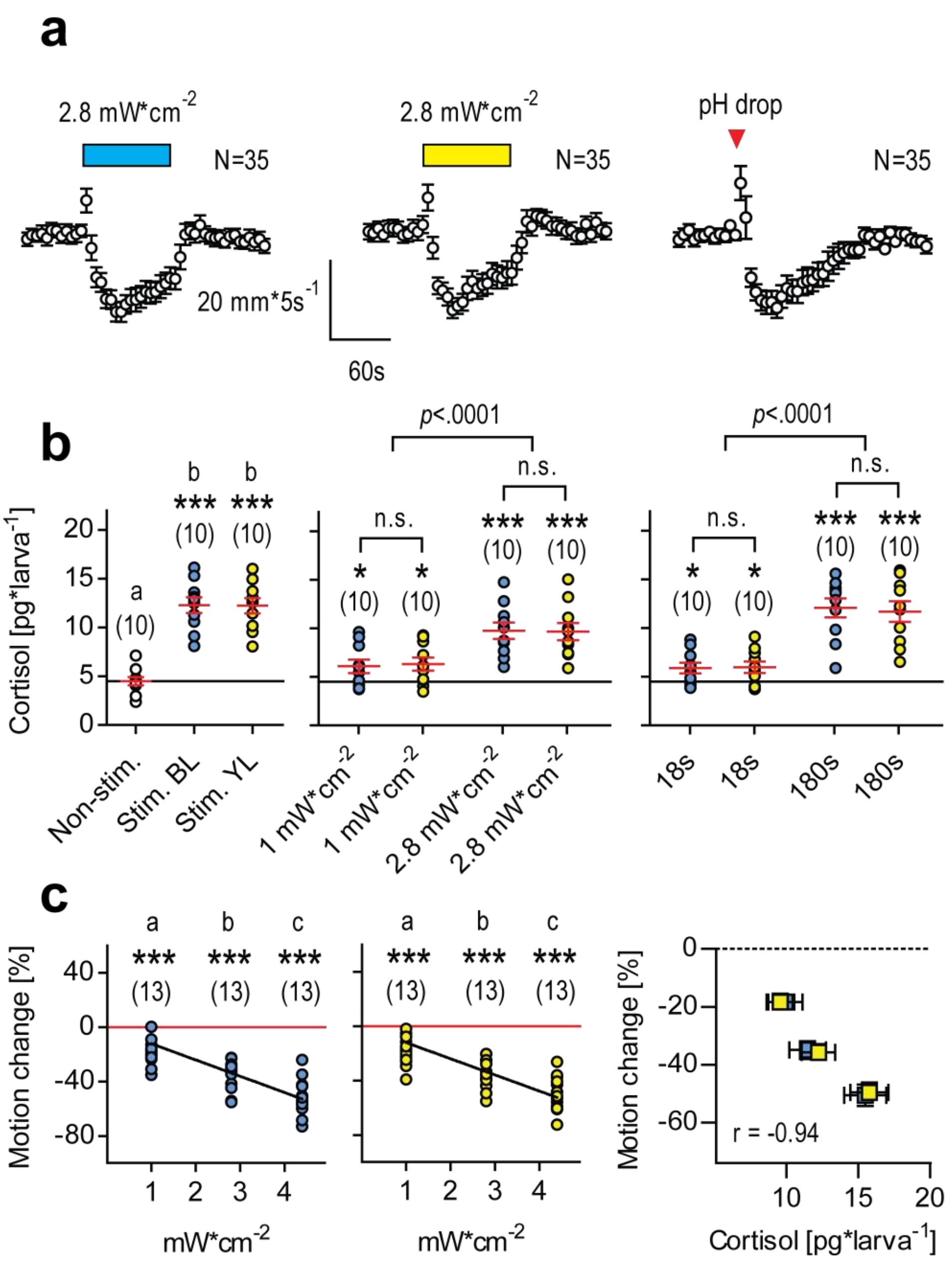

Supplementary Figure 1 | **Light acts as a potent stressor**. **a**, Left, Center, briefly dark-adapted larvae react to a squared pulse of either blue (left) or yellow (center) light with reduced and increased locomotion after the onset and offset of light, respectively. Right, they react similarly to a pH drop, a known potent stressor in fish. **b**, A squared pulse of light can increase whole-body cortisol (left, light power: 4.4 mW*cm^{-2}), depending on light power (center) and exposure time (right, light power: 2.8 mW*cm^{-2}). Left, One-Way ANOVA,

$F(2,29) = 42.1$, $p < 0.0001$, followed by Bonferroni's tests. Center, Two-Way ANOVA, Wavelength factor: $F(1,36) = 0.01$, $p = 0.93$, Light Power factor: $F(1,36) = 19.8$, $p < 0.0001$, Wavelength x Light Power factor: $F(1,36) = 0.03$, $p = 0.86$, followed by post hoc comparisons. Right, Two-Way ANOVA, Wavelength factor: $F(1,36) = 0.03$, $p = 0.87$, Length factor: $F(1,36) = 52.1$, $p < 0.0001$, Wavelength x Length factor: $F(1,36) = 0.08$, $p = 0.79$, followed by post hoc comparisons. *$p < 0.05$, ***$p < 0.001$ after one sample t-tests against basal cortisol in non-stimulated larvae (black line), Non-stim: $t(9) = 0.01$, $p = 0.99$, Stim. BL: $t(9) = 9.6$, $p < 0.0001$, Stim. YL: $t(9) = 9.9$, $p < 0.0001$, 1 $mW*cm^{-2}$ BL: $t(9) = 8.8$, $p < 0.0001$, 1 $mW*cm^{-2}$ YL: $t(9) = 9.3$, $p < 0.0001$, 2.8 $mW*cm^{-2}$ BL: $t(9) = 11.4$, $p < 0.0001$, 2.8 $mW*cm^{-2}$ YL: $t(9) = 10.9$, $p < 0.0001$, 18 s BL, $t(9) = 2.4$, $p = 0.04$, 18 s YL, $t(9) = 2.5$, $p = 0.04$, 180 s BL, $t(9) = 7.7$, $p < 0.0001$, 180 s YL, $t(9) = 6.8$, $p < 0.0001$). **c**, Left, Center, blue (left) and yellow (center) light-mediated motion change [in %] as a function of light power, calculated as locomotion after the light onset relative to baseline locomotion, with locomotion measured as the integral of swim velocity $[mm*(10\ ms)^{-1}]$ over 120 s (One-Way ANOVA, followed by Bonferroni's tests, BL, $F(2,38) = 26.9$, $p < 0.0001$, YL, $F(2,38) = 26.2$, $p < 0.0001$. ***$p < 0.001$ after one sample t-tests against zero motion change, BL: 1 $mW*cm^{-2}$: $t(12) = 7.4$, $p < 0.0001$, 2.8 $mW*cm^{-2}$: $t(12) = 11.6$, $p < 0.0001$, 4.4 $mW*cm^{-2}$: $t(12) = 13.7$, $p < 0.0001$, YL: 1 $mW*cm^{-2}$: $t(12) = 6.4$, $p < 0.0001$, 2.8 $mW*cm^{-2}$: $t(12) = 13.0$, $p < 0.0001$, 4.4 $mW*cm^{-2}$: $t(12) = 14.1$, $p < 0.0001$, black lines correspond to linear regressions). Right, motion change (from left and center) plotted against the light-induced cortisol level (Spearman's rank correlation, $p = 0.02$).

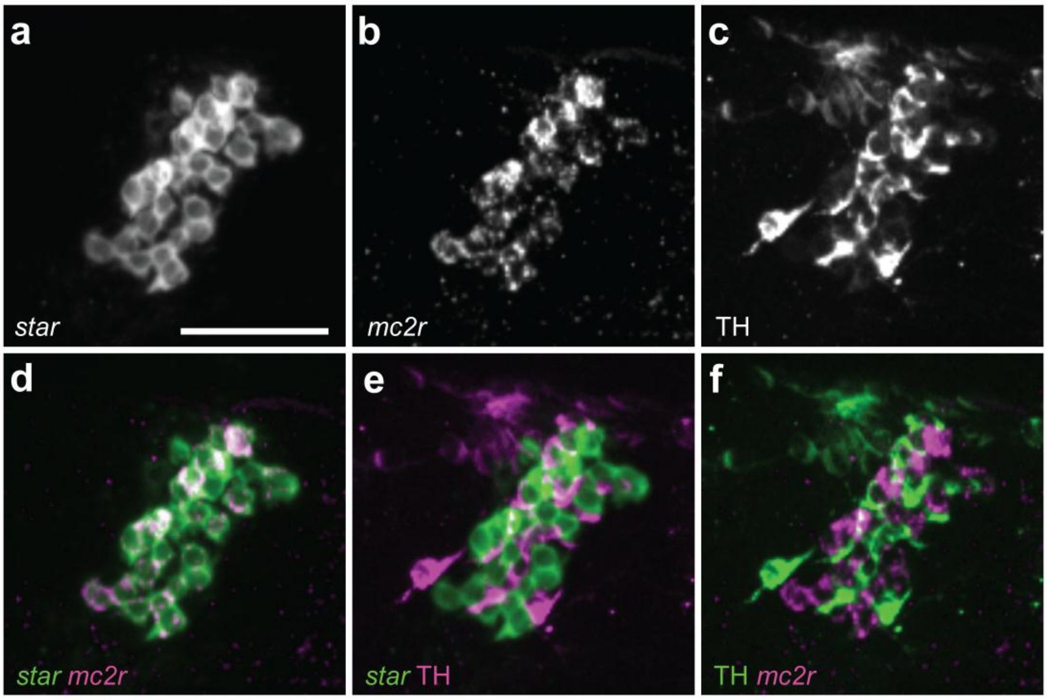

Supplementary Figure 2 | **The interrenal gland in zebrafish larvae**. **a-c**, The interrenal gland consists of steroidogenic cells expressing *star* (**a**) and the ACTH-binding *mc2r* (**b**), as shown by fluorescence *in situ* hybridization, and catecholaminergic chromaffin cells expressing TH (**c**), as shown by immunohistochemistry. **d-f**, The *star*-expressing steroidogenic cells co-express *mc2r* (**d**), and are intermingled with the TH-expressing chromaffin cells (**e**), which do not co-express *mc2r* (**f**). Scale bar: 50 µm.

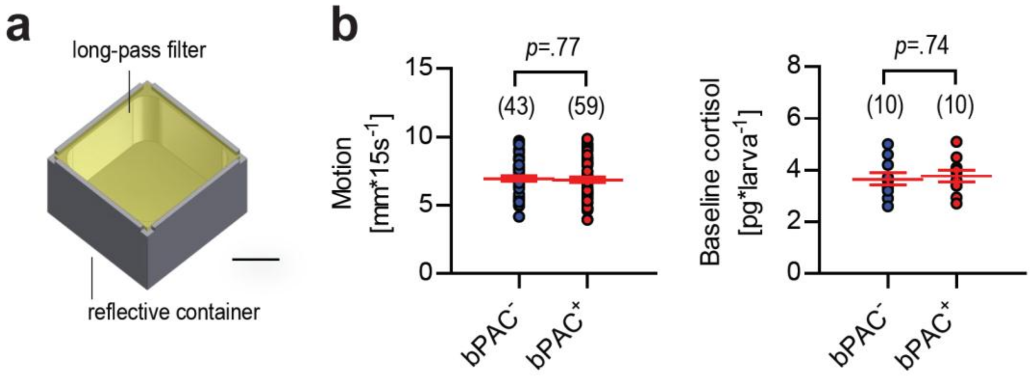

Supplementary Figure 3 | **bPAC$^+$ and bPAC$^-$ larvae have similar levels of baseline locomotion and whole-body cortisol. a**, Although bPAC has a lower dark activity than previous versions of the enzyme (1-3), we raised transgenic embryos inside reflective containers with long-pass filters to prevent light-elicited bPAC activity prior to testing. Shown is a 3D representation of a reflective container with a long-pass filter. Scale bar, 20 mm. **b**, Prior to the tests, bPAC$^+$ and bPAC$^-$ larvae showed similar levels of baseline locomotion (Two-tailed t-test, $t(100) = 0.3$, $p = 0.77$) and whole-body cortisol (Two-tailed t-test, $t(18) = 0.3$, $p = 0.74$). Sample size in parentheses. See also (4).

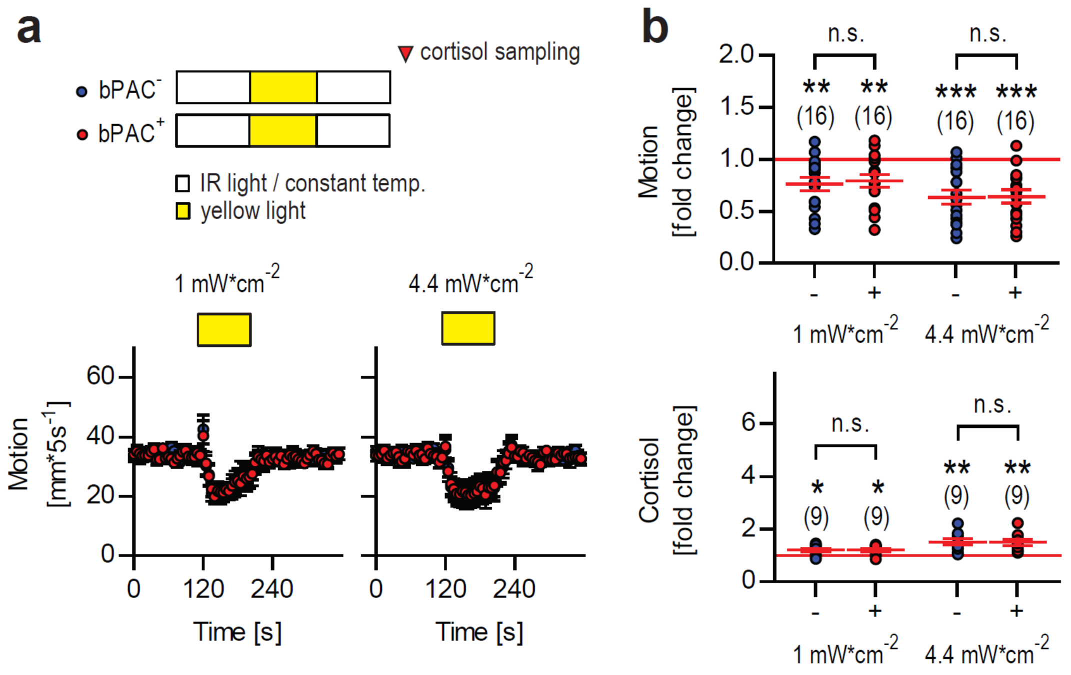

Supplementary Figure 4 | **Endocrine and locomotor reactions to yellow light in bPAC^{+} and bPAC^{-} larvae**. **a**, Top, groups and protocol for testing locomotor and cortisol reactions of bPAC^{+} and bPAC^{-} larvae to yellow light. Bottom, individual motion (mean ± s.e.m., in mm*(5 s)$^{-1}$) before, during and after a squared pulse of yellow light of either low (left) or high (right) power. **b**, Light-mediated motion (top) and cortisol (bottom) change in bPAC^{+} and bPAC^{-} larvae as a function of light power. *p < 0.05, **p < 0.01, ***p < 0.001 after one sample t-tests against a fold change of 1 (red lines), top, 1 mW*cm^{-2}, bPAC^{-}: t(15) = 3.7, p = 0.002, bPAC^{+}: t(15) = 3.3, p = 0.005, 4.4 mW*cm^{-2}, bPAC^{-}: t(15) = 5.4, p < 0.0001, bPAC^{+}: t(15) = 5.7, p < 0.0001, bottom, 1 mW*cm^{-2}, bPAC^{-}: t(8) = 2.9, p = 0.02, bPAC^{+}: t(8) = 3.2, p = 0.01, 4.4 mW*cm^{-2}, bPAC^{-}: t(8) = 4.0, p = 0.004, bPAC^{+}: t(8) = 4.2, p = 0.003. 'n.s.' indicates results of Bonferroni's tests after a Two-Way ANOVA, top, Genotype factor: F(1,60) = 0.09, p = 0.76, Light Power factor: F(1,60) = 4.7, p = 0.04, Genotype x Light Power factor: F(1,60) = 0.03, p = 0.87, bottom, Genotype factor: F(1,32) = 0.01, p = 0.92, Light Power factor: F(1,32) = 9.1, p = 0.005, Genotype x Light Power factor: F(1,32) = 0.01, p = 0.94. Sample size in parentheses.

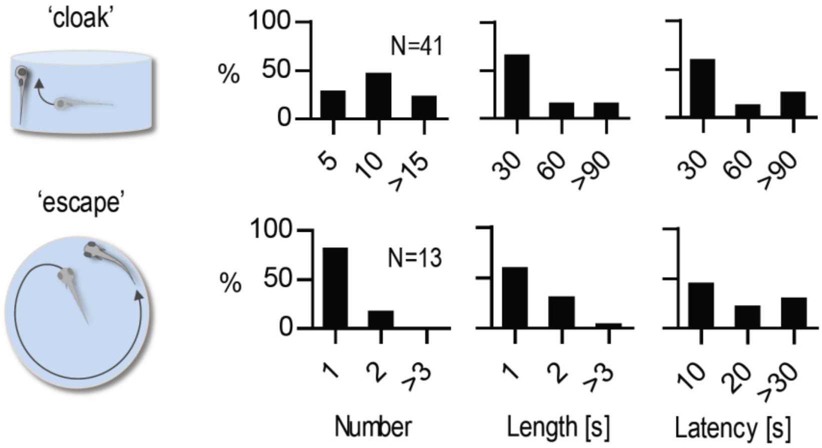

Supplementary Figure 5 | **Cloak and escape in wild-type larvae.** Left, Scheme illustrating both behaviors as elicited by continuous light exposure: ‘cloak’ (top), negative geotaxis combined with thigmotaxis, and ‘escape’ (bottom), a swimming burst. Right, Distributions of the number, length and latency of cloak and escape reactions during blue light stimulation. Light power, 2.8 mW*cm^{-2}.

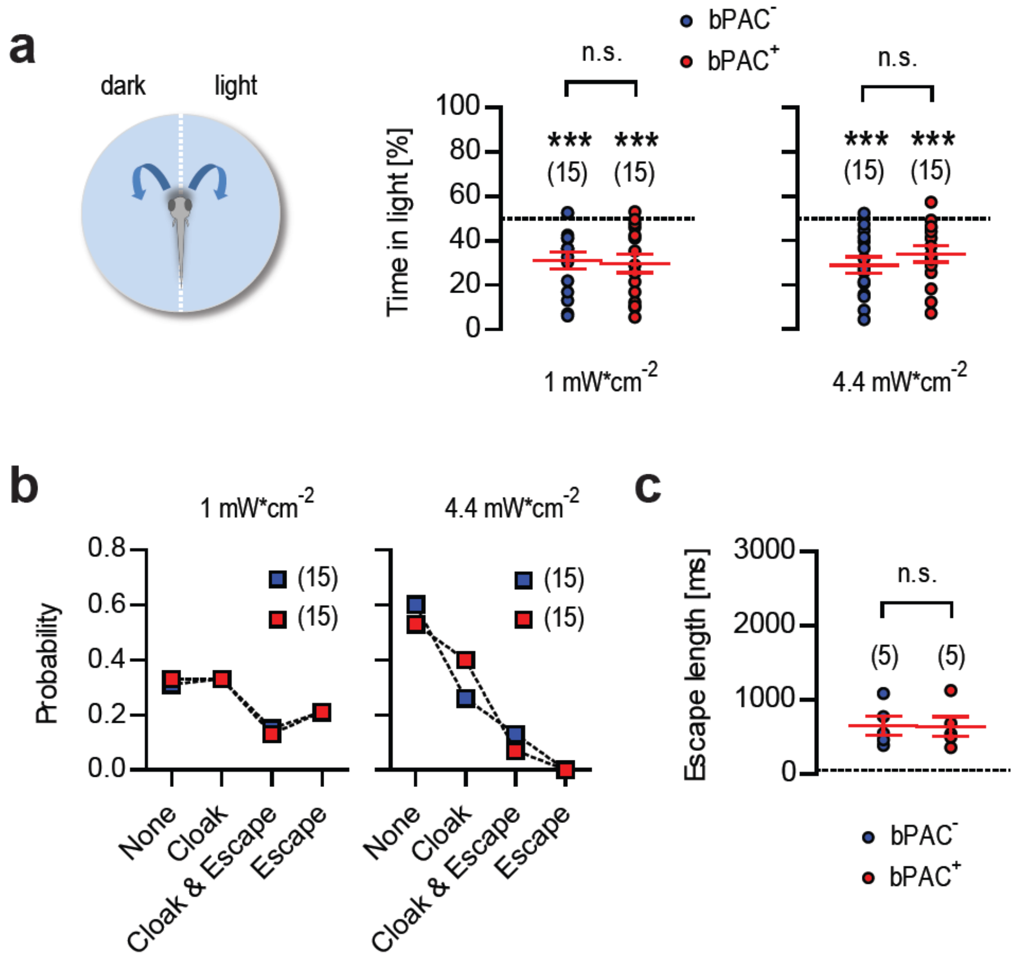

Supplementary Figure 6 | **Stressor avoidance in bPAC siblings in response to yellow light**. **a**, Left, configuration for assessing light avoidance in freely swimming larvae (as in Fig. 2a). Right, cumulative time spent under yellow light by individual $bPAC^+$ (red) and $bPAC^-$ (blue) larvae over a 120 s illumination period [in %] relative to a maximum of 120 s, for two different light powers; 'n.s.' shows results from Two-tailed t-tests (1 $mW*cm^{-2}$: $t(28) = 0.3$, $p = 0,81$, 4.4 $mW*cm^{-2}$: $t(28) = 1.0$, $p = 0,33$). ***$p < 0.001$ after one sample t-tests against 50 % (dashed lines), 1 $mW*cm^{-2}$, $bPAC^-$: $t(14) = 4.9$, $p = 0.0002$, $bPAC^+$: $t(14) = 4.8$, $p = 0.0003$, 4.4 $mW*cm^{-2}$, $bPAC^-$: $t(14) = 5.6$, $p < 0.0001$, $bPAC^+$: $t(14) = 4.4$, $p = 0.0006$. **b**, Probabilities of responses to yellow light in $bPAC^+$ and $bPAC^-$ larvae as a function of light power. **c**, Length of escape reactions by individual $bPAC^+$ and $bPAC^-$ larvae during yellow light exposure (Two-tailed t-test, $t(8) = 0.06$, $p = 0.95$). Light power, 1 and 4.4 $mW*cm^{-2}$. Sample size in parentheses.

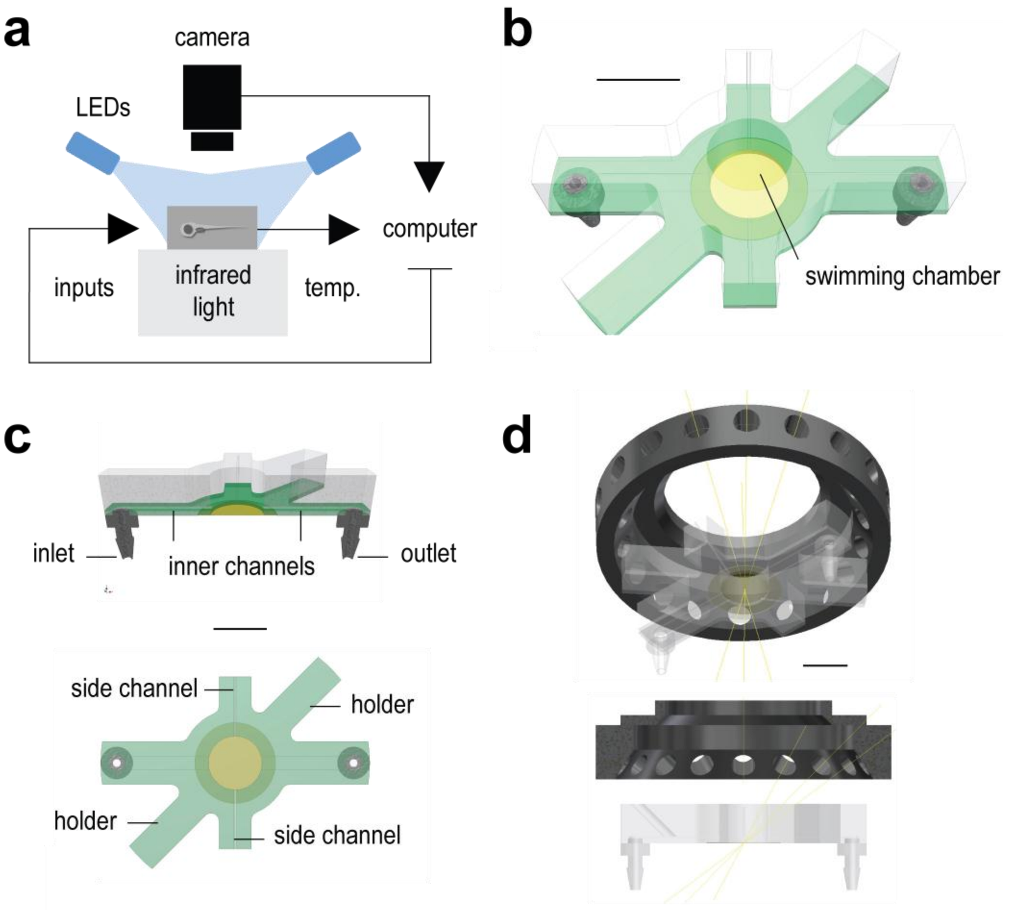

Supplementary Figure 7 | **Setup**. **a**, Configuration for imaging zebrafish larvae in combination with light stimulation and behaviorally relevant stimuli. Modified from (5). **b**, **c**, Swimming chamber (**b**) with inner channels (**c**) allowing medium to flow at constant or increasing temperature; side channels hold a thermocouple and a capillary tube producing subtle water motions. Scale bar, 10 mm. **d**, LED ring for blue and yellow light stimulation in combination with video tracking. Scale bar, 10 mm. Modified from (5).

SUPPLEMENTARY DISCUSSION

Larval zebrafish as a model system for stress research | Adult zebrafish show increased cortisol levels and stress reactions upon stressor exposure (6-11). Larval zebrafish also respond to stressors with increased whole-body cortisol (11-21). Their basal level of whole-body cortisol and expression levels of genes responsible for corticosteroid synthesis and signaling increase drastically around the time of hatching, uncovering an HPI axis that matures very early in development from 4 days post fertilization (dpf) onwards (12-14). Moreover, interactions of GRs and serotonin signaling are present in zebrafish too (22, 23). Furthermore, stressors cause avoidance reactions and induce predictable alterations in the locomotor activity and the feeding drive of freely swimming larvae (18, 19). Also, the preoptic nucleus in teleosts is considered a structure homologous to the mammalian PVN (24, 25) and the larvae's neurosecretory preoptic-hypothalamic area (NPO) is homologous to the hypothalamic paraventricular nucleus in mammals (26). Innervation of the pituitary by NPO cells is established by 3 dpf (20, 26). Oxytocin, specifically synthesized in NPO cells, has a proangiogenic function and is required for the formation of pituitary vascularization (27); pharmacological ablation of NPO cells impairs pituitary innervation and causes stressed larvae to show reduced cortisol production as compared to control larvae (20). In addition, ablation and activation of cortisol-producing interrenal cells reduces and increases whole-body cortisol upon stressor exposure, respectively (21). Two-photon calcium imaging on intact larvae shows that the magnitude of the response of individual NPO CRH cells to stressors co-varies with stressor intensity, and that stressor exposure leads to the recruitment of previously inactive CRH neurons in an intensity-dependent manner, thus increasing the pool of responsive CRH cells. This stressor-mediated activity of CRH neurons is highly synchronized (28). Most importantly, due to their small size, genetic amenability and

transparent body, larval zebrafish are suitable for high-throughput behavioral genetics, non-invasive brain imaging and optogenetic probing of neural circuits (29, 30). Notably, all three elements of the HPI axis can be visualized and manipulated using genetic tools (Supplementary Movie 1).

Non-invasive monitoring of pituitary corticotroph cell activity | Although direct measures of larval ACTH levels are far outside the range of detection thresholds of commercial ELISA kits (31), pituitary corticotrophs show a sustained elevation of intracellular Ca^{2+} upon a CRH-evoked depolarization due to an extracellular Ca^{2+} entry via voltage-gated Ca^{2+} channels (VGCC) (32). CRH causes the closure of background TWIK-related K^{+} (TREK-1) channels via a cAMP/PKA pathway and the resulting depolarization activates this extracellular VGCC Ca^{2+} entry (33). ACTH release is initiated by an intracellular Ca^{2+} release and voltage-gated extracellular Ca^{2+} entry, together increasing local Ca^{2+} concentrations near exocytic sites (34). Other ACTH secretagogues also elevate intracellular Ca^{2+} through different mechanisms; in AtT-20 cells, CRH as well as cAMP cause intracellular Ca^{2+} elevation and cAMP enhanced VGCC (35, 36).

SUPPLEMENTARY METHODS

Supplementary Movie 1 | Cell populations forming the three elements of the HPI axis were reconstructed using specific transgenic expression in stable lines and *in situ* cell type mapping. Transgenic larvae expressing *OtpECR6:RFPcaax* (20), *pomc:GFP* (37) and *StAR:GFP* (21) were imaged *in vivo* to reconstruct the position of hypothalamic NPO cells (blue), pituitary *pomc*-expressing cells (red), and interrenal *star*-expressing cells (green) using

spherical labels in Amira's segmentation editor. Skin autofluorescence of multiple animals imaged dorsally and laterally was used to reconstruct and register the outline of the fish (gray). Cell position reconstructions from *in situ* mapping experiments (26) were registered into the position of the NPO indicated by *OtpECR6:RFPcaax* expression.

SUPPLEMENTARY REFERENCES

1. Schroder-Lang, S. et al. Fast manipulation of cellular cAMP level by light *in vivo*. *Nat. Methods* **4,** 39–42 (2007).

2. Ryu, M. H., Moskvin, O. V., Siltberg-Liberles, J., Gomelsky, M. Natural and engineered photoactivated nucleotidyl cyclases for optogenetic applications. *J. Biol. Chem.* **285,** 41501–41508 (2010). doi: 10.1074/jbc.M110.

3. Stierl, M. et al. Light modulation of cellular cAMP by a small bacterial photoactivated adenylyl cyclase, bPAC, of the soil bacterium *Beggiatoa*. *J. Biol. Chem.* **286,** 1181–1188 (2011). doi: 10.1074/jbc.M110.185496.

4. De Marco, R. J., Groneberg, A. H., Yeh, C. M., Castillo Ramírez, L. A., Ryu, S. Optogenetic elevation of endogenous glucocorticoid level in larval zebrafish. *Front. Neural Circuits* **7,** 82 (2013). doi: 10.3389/fncir.2013.00082.

5. Groneberg, A.H., Herget, U., Ryu, S., De Marco, R.J. Positive taxis and sustained responsiveness to water motions in larval zebrafish. *Front. Neural Circuits* **9,** 9 (2015). doi: 10.3389/fncir.2015.00009.

6. Ramsay, J. et al. Whole-body cortisol is an indicator of crowding stress in adult zebrafish, *Danio rerio*. *Aquaculture* **258,** 565–574 (2006).

7. Ramsay, J. M. et al. Whole-body cortisol response of zebrafish to acute net handling stress. *Aquaculture* **297,** 157–162 (2009).

8. Speedie, N., Gerlai, R. Alarm substance induced behavioral responses in zebrafish (*Danio rerio*). *Behav. Brain Res*. **188,** 168–177 (2008).

9. Egan, R. J. et al. Understanding behavioral and physiological phenotypes of stress and anxiety in zebrafish. *Behav. Brain Res*. **205,** 38–44 (2009).

10. Cachat, J., et al. Measuring behavioral and endocrine responses to novelty stress in adult zebrafish. *Nat. Protoc*. **5,** 1786–1799 (2010).

11. Steenbergen, P. J., Richardson, M. K., Champagne, D. L. The use of the zebrafish model in stress research. *Prog. Neuropsychopharmacol. Biol. Psychiatry* **35,** 1432–1451 (2011).

12. Alsop, D., Vijayan, M. M. Development of the corticosteroid stress axis and receptor expression in zebrafish. *Am. J. Physiol. Regul. Integr. Comp. Physiol*. **294,** R711–R719 (2008).

13. Alsop, D., Vijayan, M. M. Molecular programming of the corticosteroid stress axis during zebrafish development. *Comp. Biochem. Physiol. A Mol. Integr. Physiol.* **153,** 49-54 (2009).

14. Alderman, S. L., Bernier, N. J. Ontogeny of the corticotropin-releasing factor system in zebrafish. *Gen. Comp. Endocrinol*. **164,** 61–69 (2009).

15. Yeh, C. M., Glock, M., Ryu, S. An optimized whole-body cortisol quantification method for assessing stress levels in larval zebrafish. *PLoS One* **8,** e79406 (2013). doi: 10.1371/journal.pone.0079406.

16. Fuzzen, M. L., Van Der Kraak, G., Bernier, N. J. Stirring up new ideas about the regulation of the hypothalamic-pituitary-interrenal axis in zebrafish (*Danio rerio*). *Zebrafish* **7,** 349–358 (2010).

17. Clark, K. J., Boczek, N. J., Ekker, S. C. Stressing zebrafish for behavioral genetics. *Rev. Neurosci*. **22,** 49–62 (2011).

18. De Marco, R. J., Groneberg, A. H., Yeh, C. M., Castillo-Ramírez, L.A., Ryu, S. Optogenetic elevation of endogenous glucocorticoid level in larval zebrafish. *Front. Neural Circuits* **7,** 82 (2013).

19. De Marco, R. J., Groneberg, A. H., Yeh, C. M., Trevino, M., Ryu, S. The behavior of larval zebrafish reveals stressor-mediated anorexia during early vertebrate development. *Front. Behav. Neurosci.* **8,** 367 (2014).

20. Gutierrez-Triana, J. A., Herget, U., Lichtner, P., Castillo-Ramírez, L. A., Ryu, S. A vertebrate-conserved *cis*-regulatory module for targeted expression in the main hypothalamic regulatory region for the stress response. *BMC Dev. Biol.* **14,** 41 (2014). doi: 10.1186/s12861-014-0041-x.

21. Gutierrez-Triana, J. A., et al. Manipulation of interrenal cell function in developing zebrafish using genetically targeted ablation and an optogenetic tool. *Endocrinology* **156,** 3394-3401 (2015). doi: 10.1210/EN.2015-1021.

22. Griffiths, B. B., et al. A zebrafish model of glucocorticoid resistance shows serotonergic modulation of the stress response. *Front. Behav. Neurosci*. **6,** 68 (2012). doi: 10.3389/fnbeh.2012.00068.

23. Ziv, L., et al. An affective disorder in zebrafish with mutation of the glucocorticoid receptor. *Mol. Psychiatry* **18,** 681-691 (2012). doi: 10.1038/mp.2012.64.

24. Peter, R. E. The preoptic nucleus in fishes: a comparative discussion of function-activity relationships. *Amer. Zool*. **17,** 775–785 (1977).

25. Forlano, P. M., Cone, R. D. Conserved neurochemical pathways involved in hypothalamic control of energy homeostasis. *J. Comp. Neurol*. **505,** 235–248 (2007).

26. Herget, U., Wolf, A., Wullimann, M.F., Ryu, S. Molecular neuroanatomy and chemoarchitecture of the neurosecretory preoptic-hypothalamic area in zebrafish larvae. *J. Comp. Neurol.* **522,** 1542-1564 (2014). doi: 10.1002/cne.23480.

27. Gutnick, A. et al. The hypothalamic neuropeptide oxytocin is required for formation of the neurovascular interface of the pituitary. *Dev. Cell* **21,** 642-654 (2011).

28. vom Berg-Maurer, C., Trivedi, C., Bollmann, J., De Marco, R. J., Ryu, S. The severity of acute stress is represented by increased synchronous activity and recruitment of hypothalamic CRH neurons. *J. Neurosci.* **36,** 3350-3362 (2016). doi: 10.1523/JNEUROSCI.3390-15.2016.

29. Gahtan, E., Baier, H. Of lasers, mutants, and see-through brains: functional neuroanatomy in zebrafish. *J. Neurobiol*. **59,** 147–161 (2004).

30. Portugues, R., Severi, K. E., Wyart, C., Ahrens, M. B. Optogenetics in a transparent animal: circuit function in the larval zebrafish. *Curr. Opin. Neurobiol*. **23,** 119–126 (2013).

31. Sumpter, J. P., Dye, H. M., Benfey, T. J. The effects of stress on plasma ACTH, α-MSH, and cortisol levels in salmonid fishes. *Gen. Comp. Endocrinol*. **62,** 377-385 (1986).

32. Lee, A. K., Tse, A. Mechanism underlying corticotropin-releasing hormone (CRH) triggered cytosolic Ca2+ rise in identified rat corticotrophs. *J. Physiol.* **504,** 367-378 (1997).

33. Tse, A., Lee, A. K., Tse, F. W. Ca2+ signaling and exocytosis in pituitary corticotropes. *Cell Calcium.* **51,** 253-259 (2012). doi: 10.1016/j.ceca.2011.12.007.

34. Tse, A., Lee, A. K. Voltage-gated Ca2+ channels and intracellular Ca2+ release regulate exocytosis in identified rat corticotrophs. *J. Physiol.* **528,** 79-90 (2000).

35. Guild, S., Reisine, T. Molecular mechanisms of corticotropin-releasing factor stimulation of calcium mobilization and adrenocorticotropin release from anterior pituitary tumor cells. *J. Pharmacol. Exp. Ther.* **241,** 125-130 (1987).

36. Luini, A., Lewis, D., Guild, S., Corda, D., Axelrod, J. Hormone secretagogues increase cytosolic calcium by increasing cAMP in corticotropin-secreting cells. *Proc. Natl. Acad. Sci. U. S. A.* **82,** 8034-8038 (1985).

37. Liu, N.A., et al. Pituitary corticotroph ontogeny and regulation in transgenic zebrafish. *Mol. Endocrinol.* **17,** 959-966 (2003).
